# The Prognostic Value of Pulmonary Venous Flow Reversal in Patients with Significant Degenerative Mitral Regurgitation

**DOI:** 10.3390/jcdd10020049

**Published:** 2023-01-28

**Authors:** Alon Shechter, Steele C. Butcher, Robert J. Siegel, Jenan Awesat, Merry Abitbol, Mordehay Vaturi, Alex Sagie, Ran Kornowski, Yaron Shapira, Idit Yedidya

**Affiliations:** 1 Department of Cardiology, Rabin Medical Center, Petach Tikva 4941492, Israel; 2 Faculty of Medicine, Tel Aviv University, Tel Aviv 69978, Israel; 3 Department of Cardiology, Smidt Heart Institute, Cedars-Sinai Medical Center, Los Angeles, CA 90048, USA; 4 Department of Cardiology, Royal Perth Hospital, Perth, WA 6000, Australia; 5 David Geffen School of Medicine, University of California Los Angeles, Los Angeles, CA 90095, USA

**Keywords:** degenerative mitral regurgitation, pulmonary veins, atrial fibrillation, prognosis

## Abstract

*Background*: The prognostic significance of pulmonary venous (PV) flow reversal in degenerative mitral regurgitation (dMR) is not well-established. *Objective*: We aimed to assess whether reversed PV flow is associated with adverse outcomes in patients with significant dMR. *Methods*: We retrospectively analyzed consecutive patients referred to a tertiary center for evaluation of dMR of greater than moderate degree, who had normal sinus rhythm, had a left ventricular ejection fraction of above 60%, and did not suffer from any other major valvular disorders. The primary outcome was the combined rate of all-cause mortality, mitral intervention, or new-onset atrial fibrillation (AF) at 5 years following index echocardiogram. Secondary outcomes included individual components of the primary outcome. *Results*: Overall, 135 patients (median age 68 (IQR, 58–74) years; 93 (68.9%) males; 89 (65.9%) with severe MR) met the inclusion criteria and were followed for 115.2 (IQR, 60.0–155.0) months. Patients with a reversed PV flow pattern (PVFP) (n = 34) more often presented with severe MR compared to those with a normal (n = 49) and non-reversed PVFP (n = 101) (RR = 2.03 and 1.59, respectively, all *p* < 0.001). At 5 years, they experienced the highest cumulative incidence of the primary outcome (80.2% vs. 59.2% and 67.3%, *p* = 0.008 and 0.018, respectively). Furthermore, a reversed PVFP was independently associated with a higher risk of the primary outcome compared to normal PVFP (HR 2.53, 95% CI 1.21–5.31, *p* = 0.011) and non-reversed PVFP (HR 2.14, 95% CI 1.12–4.10, *p* = 0.022). Conclusion: PV flow reversal is associated with a worse 5-year composite of mortality, mitral intervention, or AF in patients with significant dMR.

## 1. Introduction

Degenerative mitral regurgitation (dMR) is the second-most common form of chronic mitral regurgitation (MR) in developed nations [1]. Affecting 15 out of every 1000 adult Americans in the year 2000 [1], the disease constitutes a significant health and economic burden at an advanced stage. Timely valvular intervention is therefore of paramount importance. Previous retrospective studies have identified atrial fibrillation (AF) and left atrial (LA) dilation as predictors of increased mortality among dMR patients, both conservatively managed [2,3] and surgically treated [3,4]. Serving as the mechanistic link between MR and atrial aberrations [5,6] is LA remodeling, the manifestations of which may include abnormal pulmonary venous (PV) flow pattern (PVFP). Already considered to be supportive evidence of severe MR [7], a reversed PVFP likely signifies a worse disease state which may adversely affect prognosis. Using a contemporary cohort of real-world patients, we assessed whether pulmonary venous flow reversal was associated with worse clinical outcomes in patients with significant chronic dMR.

## 2. Methods

### 2.1. Study Population and Outcomes

This is a single-center, retrospective analysis of consecutive patients with dMR of greater than moderate grade who were referred to transesophageal echocardiography (TEE) at Rabin Medical Center (RMC), Israel, between May 1995 and June 2017.

Inclusion criteria comprised the following: 1. absence of documented AF or flutter prior to the TEE; 2. normal left ventricular (LV) systolic function, defined as an LV ejection fraction (LVEF) of above 60% on the transthoracic echocardiogram (TTE) part of the index examination; and 3. an isolated degenerative mitral pathology not accompanied by other valvular disorders of greater than mild-to-moderate grade. Patients that underwent any previous valvular intervention, as well as those without complete baseline data (importantly, the documentation of PVFP bilaterally) were excluded. Follow-up duration spanned the timeframe between the date of echocardiogram and either death or 30 April 2020.

The primary outcome was defined as the composite of all-cause mortality, any form of invasive mitral intervention, and new-onset AF or flutter during the first 5 years of follow-up, excluding arrhythmic events in the first post-interventional month. Secondary outcomes included single components of the primary outcome.

Data regarding endpoints were retrieved using Ofek Software (dbMotion, Pittsburg, PA, USA), which is a web-based medical chart platform shared by most of Israel’s public medical providers. Atrial arrythmias were ascertained by inspecting patient files for any mention of obvious AF or flutter on a 12-lead electrocardiogram (ECG) and/or an irregular ventricular electrical activity without discernible P waves on a 30-s strip [8].

The study was conducted in accordance with the Declaration of Helsinki and received Institutional Review Board (IRB) approval. The requirement for informed consent was waived due to the study’s retrospective nature.

### 2.2. Echocardiographic Assessment

**Figure 1:**
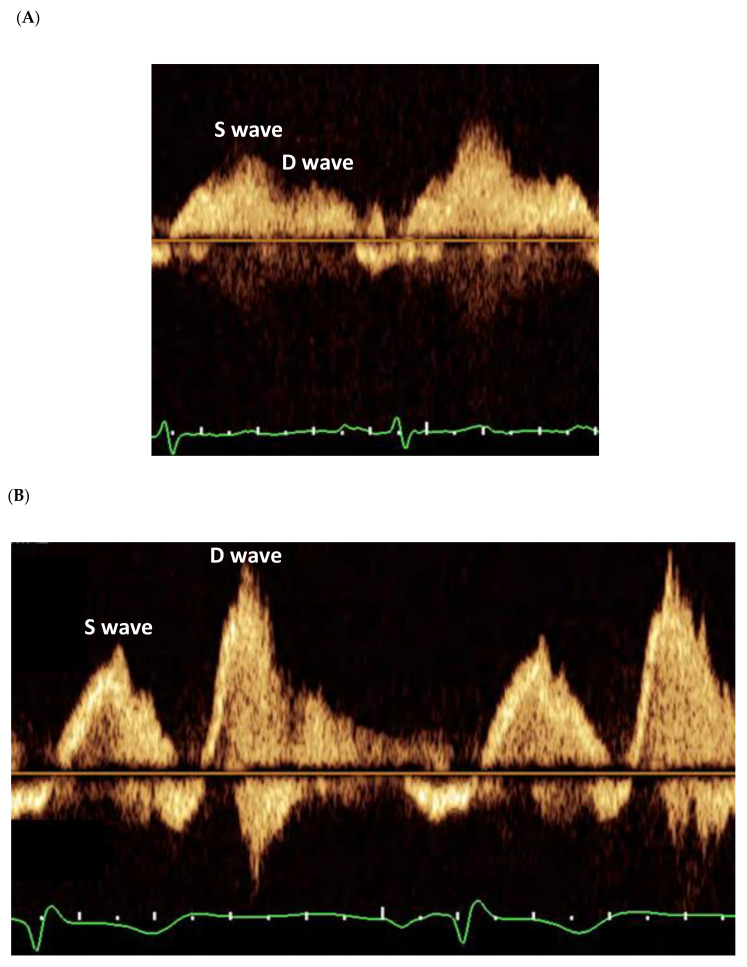

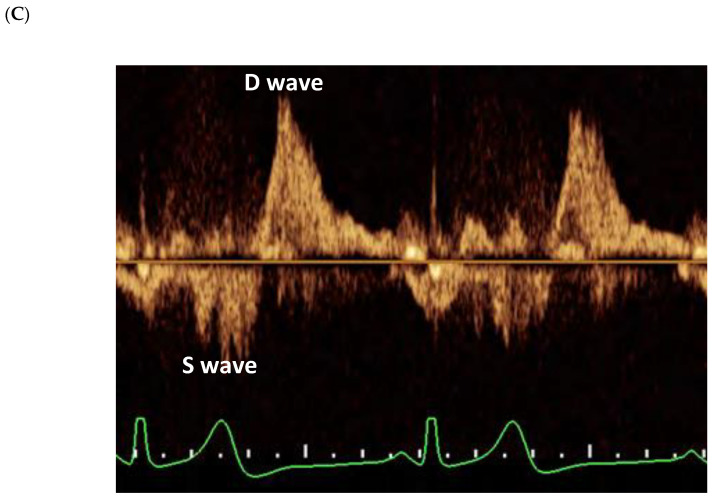
Pulmonary Venous Flow Patterns as Imaged on Transesophageal Echocardiogram. Normal pattern is signified by a peak systolic (S) to peak diastolic (D) flow velocities ratio of ≥1. Abnormal pattern may include either a blunted or a reversed S wave, manifested by an S/D ratio of <1 or <0, respectively. (**A**) Normal Flow Pattern. (**B**) Blunted Flow Pattern. (**C**) Reversed Flow Pattern.

All echocardiograms were performed and interpreted by level-III-trained sonographers and echocardiologists. Echo systems used included Vivid 3 Premium and Vivid E95 (General Electric, Milwaukee, WN, USA), Agilent 5500 (Agilent, Santa Clara, CA, USA), HP 77020 (Hewlett Packard, Andover, MA, USA), and IE33 and EPIQ (Philips, Amsterdam, The Netherlands). Diagnostic measurements and conclusions made at each study were based on criteria set forth by the relevant American Society of Echocardiography guidelines [8,9]. Mitral valve assessment incorporated standard multiple views. Regurgitation severity was determined based on the integration of qualitative and semiquantitative measures, whenever possible. A diagnosis of degenerative mitral valve (MV) disease required the visualization of leaflet prolapse, signified by a ≥2 mm atrial displacement of the leaflet tip from the mitral annular level at end-systole. Pulmonary veins (PVs) were assessed bilaterally. After verification of tangentiality by color Doppler, the flow at each PV was sampled by a pulsed-wave (PW) Doppler beam placed within 1 cm of the PV ostia. Normal PVFP was defined by a peak systolic (S) velocity to peak diastolic (D) velocity ratio of 1 and above (Figure 1); conversely, PVFP reversal was characterized by an S to D ratio of below zero. Blunted PVFP, considered a form of non-reversed PVFP, was further identified by an S to D ratio between zero and below 1. Overall flow was determined according to the lowest S to D ratio observed. Pulmonary arterial systolic pressure (PASP) assessment was based on the peak systolic pressure gradient measured across the tricuspid valve and the estimated right atrial pressure (RAP) using the diameter and respiratory collapsibility of the inferior vena cava (IVC), both during the TTE part of each study. Global right ventricular (RV) function was evaluated qualitatively. All reports were blindly scrutinized for integrity by two physicians (A.Shechter and I.Y.).

### 2.3. Statistical Analysis

The study cohort was split into three main groups according to baseline PVFP, namely normal, reversed, and non-reversed, with the latter comprising patients exhibiting either normal or blunted flow patterns in the PVs. In each group, continuous variables were expressed as means and standard deviations (SDs) or as medians and interquartile ranges (IQRs). Categorical variables were reported as frequencies and percentages. Between-group comparison of continuous variables with normal distribution was performed using Student’s t test, while that of continuous variables demonstrating non-normal distribution was performed using the Mann–Whitney U test. Categorical variables were compared using Pearson’s chi-squared test or Fisher’s exact test. Importantly, only two groups were compared at a time.

The risk for the development of the primary outcome according to PVFP was graphically displayed using the Kaplan–Meier method, with comparisons of cumulative survival across strata conducted using the log rank test. To identify independent associations between the primary outcome and baseline variables, particularly different forms of PVFP, univariable Cox regression analysis was employed, after which parameters showing a *p*-value of <0.1 were integrated into a multivariable model.

Patients with missing data were censored from the relevant analyses. A two-sided *p*-value of <0.05 was considered to represent statistical significance. All analyses were performed using SPSS^TM^ Statistic for Windows software, version 24 (IBM Corporation, Armonk, NY, USA).

## 3. Results

### 3.1. Baseline Characteristics of the Study Population

**Figure 2:**
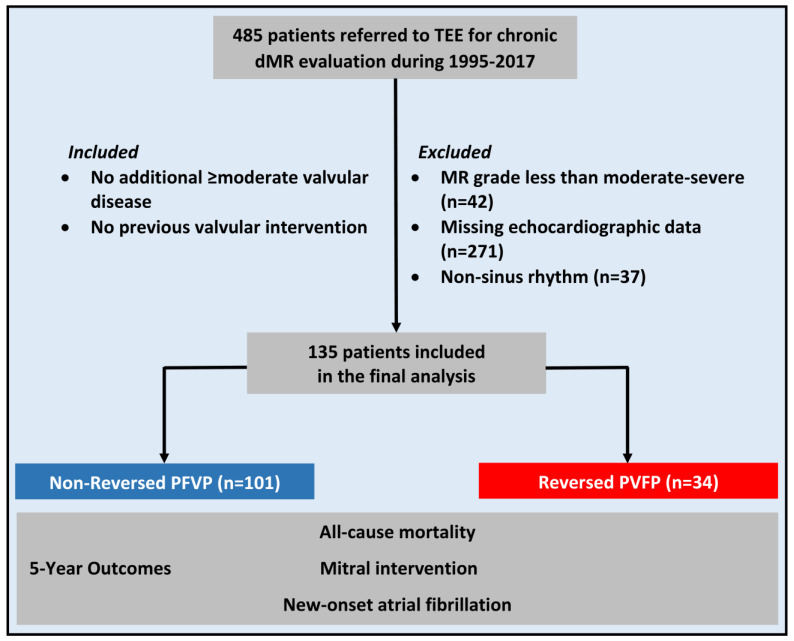
Study Flow Chart. dMR = degenerative mitral regurgitation; LV = left ventricle; NSR = normal sinus rhythm; PVFP = pulmonary venous flow pattern.

Out of 485 patients that underwent TEE for an evaluation of chronic dMR at RMC between May 1995 and June 2017, a total of 135 met the study inclusion criteria (Figure 2). The median follow-up duration was 115.2 (IQR, 60.0–155.0) months. Three patients —two in the normal PVFP group and one in the reversed PVFP group—were lost to follow-up after a median of 20.0 months.

Baseline clinical and echocardiographic characteristics of the study cohort are presented in Table 1 and Table 2, respectively. Notably, the patients were mostly male (n = 93, 68.9%) and the median age was 68 (IQR, 58–74) years. Additionally, more than half (n = 70, 56.5%) were hypertensive. MR was concluded as severe in 89 (65.9%) cases and prolapse mainly involved the posterior leaflet alone (n = 94, 69.5%), followed by both leaflets (n = 26, 19.3%) and the anterior leaflet only (n = 15, 11.1%).

### 3.2. Pulmonary Venous Flow Pattern

Overall, 49 (36.3%) patients exhibited a normal flow pattern in the PVs on both sides and 86 (63.7%) patients had an abnormal PVFP on at least one side. Among the latter, 34 (25.2%) were diagnosed with a reversed PVFP and 52 (38.5%) had a blunted PVFP. The proportion of PV flow reversal was considerably higher among individuals with severe MR compared to those with moderate-to-severe MR (31/89; 34.8% vs. 3/46; 6.5%, *p* < 0.001).

Compared to patients with normal or non-reversed PVFP, those with reversed PVFP were less likely to be obese and diabetic (Table 1). While similarly symptomatic overall (n = 19, 76.0%), they exhibited greater functional impairment, expressed by the New York Heart Association (NYHA) classification, which was statistically significant when comparing the non-reversed and reversed PV flow groups. Lastly, patients with a reversed PVFP had the highest prevalence of severe MR (91.2%), as well as increased PASP values (Table 2). No significant differences were noted in other clinical and echocardiographic parameters, including LV end-systolic diameter, LA dimensions, and prolapse site.

### 3.3. Outcomes

After 5 years of follow-up, 11 (8.1%) patients died, 87 (64.4%) underwent mitral intervention, and 22 (16.3%) developed AF or flutter (Table 3). Intervention types included, in decreasing frequency, the following: surgical repair (n = 60, 69%); surgical replacement (n = 19, 21.8%); and transcatheter edge-to-edge repair (TEER) using the MitraClip system (Abbott Vascular Inc, Santa Clara, CA, USA) (n = 8, 9.2%).

**Figure 3:**
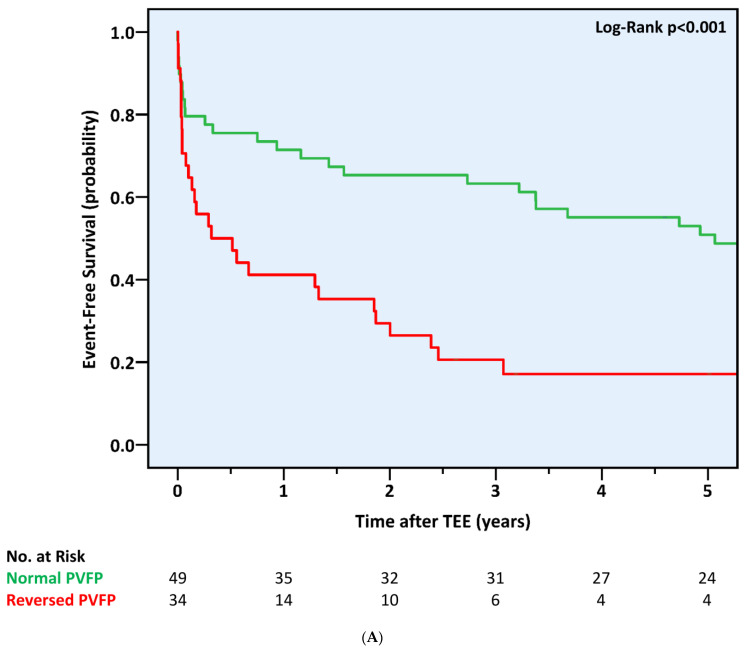

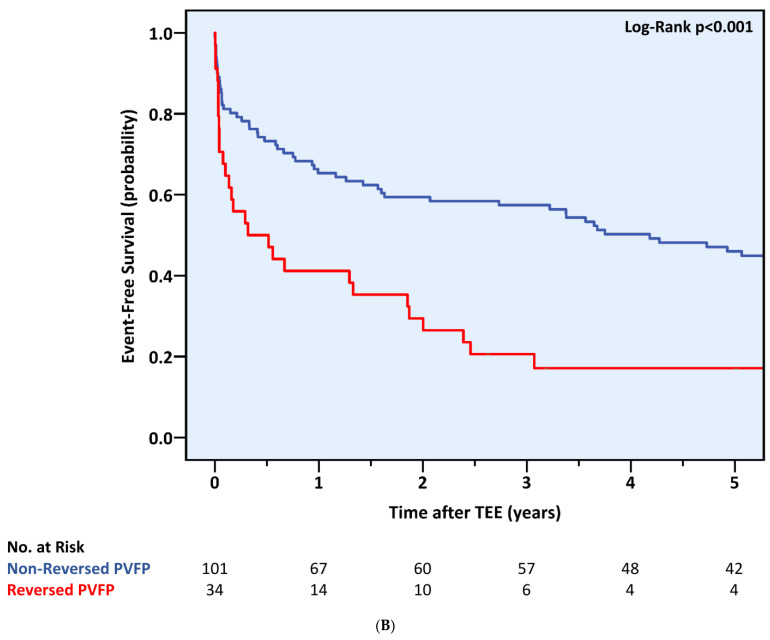
Five-Year Cumulative Incidence of the Combined Outcome of All-Cause Mortality, Mitral Intervention, or New-Onset Atrial Fibrillation According to Pulmonary Venous Flow Pattern at Baseline. PVFP = pulmonary venous flow pattern; TEE = transesophageal echocardiogram. (**A**) Normal vs. Reversed Pulmonary Venous Flow Pattern. (**B**) Non-Reversed vs. Reversed Pulmonary Venous Flow Pattern.

The primary outcome, a composite of all three separate endpoints, was reported in 98 (72.6%) patients and proved significantly more frequent in those with reversed PVFP at baseline (88.2% compared to 59.2% in the normal PVFP group and 67.3% in the non-reversed PVFP group, *p* = 0.004 and *p* = 0.018, respectively). This was reflected in significantly shorter event-free survival durations within the reversed PVFP group (16.0 ± 20.1 vs. 38.4 ± 25.9 and 34.9 ± 25.8 months, respectively, all log rank *p* < 0.001) (Figure 3). Notably, PV flow reversal was also associated with an increased cumulative incidence of the primary outcome when compared to a blunted PVFP (88.2% vs. 75.0%, *p* = 0.022). By contrast, patients with PV flow blunting experienced a non-statistically higher rate of the primary outcome compared to that of patients with a normal PVFP (75.9% vs. 59.2%, *p* = 0.137) (Supplemental Figure S1).

According to a Cox proportional hazard model, presented in Supplemental Table S1 (univariable analysis) and in Table 4 (multivariable analysis), a reversed PVFP independently predicted an increased risk for the primary outcome compared to both normal PVFP (HR 2.53, 95% CI 1.21–5.31, *p* = 0.011) and non-reversed PVFP (HR 2.14, 95% CI 1.12–4.10, *p* = 0.022).

Of the secondary outcomes, mitral interventions (but not deaths and AF or flutter) occurred earlier and more frequently during 5 years of follow-up in patients with reversed PVFP compared to patients with normal or non-reversed PVFP (Table 3). After multivariable analysis, PV flow reversal at baseline arose as an independent risk factor for mitral intervention, but not for mortality or new-onset AF (Supplemental Table S2).

## 4. Discussion

Our study examined the prognostic utility of PV flow reversal, as observed on TEE, in patients with significant dMR, normal LV systolic function, and normal sinus rhythm. Based on a retrospective, single-center analysis of 135 consecutive cases, we observed the following: 1. a reversed PVFP was evident in approximately one-quarter of the cohort and among almost all patients with severe MR; 2. its presence was associated with a more severe MR and a higher pulmonary arterial pressure, as well as with a lower functional status at baseline; 3. patients with PV flow reversal experienced a higher cumulative incidence of deaths, mitral interventions, or new-onset AF during 5 years of follow-up compared to those with either normal, non-reversed, or blunted PV flow patterns; and 4. PV flow reversal independently predicted a higher risk for mitral interventions and the composite of the three separate endpoints, more than doubling the risk for both compared to normal and non-reversed PVFP.

As a surrogate of altered LA hemodynamics [10], abnormal flow in PVs has been previously shown to manifest in disease states characterized by elevated LA filling pressures, such as MV disorders [5], AF [11], diastolic dysfunction [12], and atrioventricular (AV) dissociation [13]. In MR, a reversed PVFP could also represent a direct effect of the regurgitant jet which, depending on the exact location of the underlying pathology, may be situated against the PV ostia [14]. As both filling pressures and the regurgitant jet are not exclusively determined by the mere severity of the valvular disease, PV flow abnormalities, while highly specific, are not 100% sensitive to significant MR. This could explain the less-than 100% prevalence of reversed PVFP in our cohort, particularly among patients with severe MR.

In line with prior studies that have linked a reversed PV flow with severe MR [15], a larger proportion of patients in our study exhibiting this finding had severe MR compared to patients with a normal flow pattern, and vice versa. However, the hazardous association between reversed PVFP and outcomes remained after controlling for MR grade, suggesting that PV flow reversal may serve not only as a diagnostic criterion for severe dMR, but also as a prognostic marker in both moderate-to-severe and severe dMR. Theoretically, the worse prognosis experienced by patients with dMR and a reversed PVFP may have been not only the result of the more advanced valvular disease, but also a reflection of an accompanying atrial myopathy, a condition well-described in the literature [16,17], which could by itself lead to reduced cardiac output, elevated pulmonary vascular pressure, and thromboembolism [18]. Furthermore, patients with PV flow reversal may have experienced a more pronounced diastolic dysfunction, which also could contribute to a less favorable prognosis. While this last notion could not be fully ascertained by echocardiography due to the presence of significant MV disease, altered PVFP has previously been correlated with diastolic dysfunction using right heart catheterization [19], and in our study was indeed associated with LA dilation and pulmonary hypertension, both of which are mutually related to LA pathology and LV stiffness [20]. Still, the fact that PV flow reversal demonstrated an independent prognostic ability according to a comprehensive multivariable analysis that considered all of the above-mentioned parameters may suggest a dominant role for MR grade in that regard nonetheless.

On a final note, our findings may have therapeutic implications, particularly among dMR patients who do not fulfill current practice guideline criteria for intervention [8,21]. As mentioned, the worse 5-year outcomes observed within the reversed PVFP group encompassed both patients with severe MR and normal LV function, as well as patients with less than severe (i.e., moderate-to-severe) MR. Although regurgitation severity is also a function of loading conditions and may thus fluctuate, and while MR severity, rather than PVFP, may have been the driving force for the higher event rate in our cohort, a reversed PVFP was independently associated with the primary outcome and with mitral interventions. In view of its prognostic impact, PV flow reversal may warrant earlier intervention in patients with significant dMR. Future research may assess the implication of integrating this finding in the decision-making process when caring for dMR patients.

## 5. Limitations

A single-center, retrospective design may undermine the external validity of the study results. Nevertheless, we employed the largest cohort to date of significant dMR patients with normal LV function and sinus rhythm that were specifically assessed for PVFP. Moreover, baseline patient characteristics were apparently comparable to those presented by previous reports of dMR populations [22] and echocardiographic reports were blindly assessed. Regarding conceptual matters, death causes and mitral intervention indications, as well as additional indices of atrial myopathy (such as indexed volume and serum natriuretic peptides) and of diastolic function (including invasive hemodynamic parameters), were not explored, thus impairing the ability to identify the exact pathogenic correlations and consequences of a deranged PVFP. Additionally, AF diagnoses were mostly based on surface ECGs and not continuous tracings, the use of which could have led to more accurate estimates of arrhythmic burden. During the protracted timeframe of the study, practice guidelines changed, potentially affecting the interpretation of baseline observations and the definition of downstream events. However, all patients were assessed concomitantly and exposed to similar methodologies, arguably making any interaction with such an evolution non-significant. Lastly, echocardiographic assessments of MR and PVFP may be prone to operator-dependent errors and patient-specific features, such as hemodynamic status. Nevertheless, we noted a significant association between MR severity and PVFP anomaly and deliberately included both moderate-to-severe and severe MR cases, keeping in mind the possibility of shifting severity of the valvular disease.

## 6. Conclusions

In our single-center experience, patients with significant dMR, normal LV systolic function, and normal sinus rhythm experienced earlier, more frequent composite events of death, mitral intervention, or new-onset AF at 5 years when faced with a reversed PVFP. Furthermore, PV flow reversal was independently associated with more than twice the risk for the composite outcome compared to normal and non-reversed flow patterns, regardless of MR severity, LA diameter, or pulmonary arterial systolic pressure.

## Figures and Tables

**Table 1:** Baseline Clinical Characteristics of the Study Population.

					*p*-Value	
	Total Cohort (N = 135)	Normal PVFP (N = 49)	Reversed PVFP (N = 34)	Non-Reversed PVFP (N = 101)	Normal vs. Reversed	Non-Reversed vs. Reversed
Demographic Data						
Age (years)	68 (58–74)	68 (58–73)	69 (56–76)	68 (58–74)	0.636	0.941
Male sex	93 (68.9)	31 (63.3)	24 (64.7)	70 (70.3)	1.000	0.542
Comorbidities						
BMI						
Median (kg/m^2^)	25.0 (22.5–27.2)	25.7 (23.2–28.9)	23.1 (20.5–25.3)	26.0 (23.0–27.9)	0.007	0.001
≥30 kg/m^2^	11 (10.8)	6 (18.2)	0 (0.0)	11 (15.1)	0.026	0.027
BSA (m^2^)	1.82 (1.69–1.98)	1.80 (1.70–1.93)	1.80 (1.59–1.93)	1.85 (1.70–2.00)	0.628	0.323
Hypertension	70 (56.5)	26 (61.9)	15 (45.5)	55 (60.4)	0.155	0.137
Diabetes Mellitus	12 (9.7)	7 (16.7)	0 (0.0)	12 (13.2)	0.016	0.035
Functional Status						
NYHA Class					0.063	0.010
I	34 (34.7)	14 (37.8)	6 (24.0)	28 (38.4)		
II	38 (38.8)	14 (37.8)	8 (32.0)	30 (41.1)		
III	23 (23.5)	9 (24.3)	8 (32.0)	15 (20.5)		
IV	3 (3.1)	0 (0.0)	3 (12.0)	0 (0.0)		
II and Above	64 (65.3)	23 (62.2)	19 (76.0)	45 (61.6)	0.427	0.193
Medications						
Beta blockers	37 (30.1)	13 (31.0)	6 (18.2)	31 (34.4)	0.207	0.081
RAS inhibitors	53 (43.1)	20 (47.6)	14 (43.1)	39 (43.3)	0.654	0.928
MRAs	5 (4.1)	2 (4.8)	0 (0.0)	5 (5.6)	0.501	0.323

Data are presented as number (percentage) or median (interquartile range), where appropriate. BMI = body mass index; BSA = body surface area; MRA = mineralocorticoid receptor antagonist; NYHA = New York Heart Association; PVFP = pulmonary venous flow pattern; RAS = renin–angiotensin system.

**Table 2:** Baseline Echocardiographic Parameters.

					*p*-Value	
	Total Cohort (N = 135)	Normal PVFP (N = 49)	Reversed PVFP (N = 34)	Non-Reversed PVFP (N = 101)	Normal vs. Reversed	Non-Reversed vs. Reversed
Mitral Regurgitation						
MR Severity					<0.001	<0.001
Moderate-to-Severe	46 (34.1)	27 (55.1)	3 (8.8)	43 (42.6)		
Severe	89 (65.9)	22 (44.9)	31 (91.2)	58 (57.4)		
MR PISA EROA						
Median (cm^2^)	0.48 (0.36–0.63)	0.38 (0.30–0.49)	0.60 (0.48–0.69)	0.43 (0.33–0.54)	0.001	0.005
≥0.4 cm^2^	40 (66.7)	9 (42.9)	16 (88.9)	24 (57.1)	0.003	0.017
MR PISA RVol						
Median (mL)	71 (55–88)	60 (45–81)	81 (70–94)	68 (51–77)	0.042	0.029
≥60 mL	39 (73.6)	10 (50.0)	16 (94.1)	23 (63.9)	0.003	0.022
Prolapses Site					0.563	
Anterior	15 (11.1)	10 (20.4)	4 (11.8)	11 (10.9)	0.301	1.000
Posterior	94 (69.5)	31 (63.3)	23 (67.6)	71 (70.3)	0.681	0.771
Both	26 (19.3)	8 (16.3)	7 (20.6)	19 (18.8)	0.620	0.820
Left Heart Dimensions						
LV ESD						
Median (mm)	32 (28–37)	30 (27–37)	35 (30–39)	31 (27–37)	0.056	0.070
≥40 mm	21 (15.6)	7 (14.3)	5 (14.7)	16 (15.8)	1.000	0.874
LA Diameter						
Median (mm)	45 (40–50)	45 (40–50)	46 (40–52)	44 (40–49)	0.452	0.321
>55 mm	8 (5.9)	2 (4.1)	3 (8.8)	5 (5.0)	0.396	0.415
LA Area						
Median (cm^2^)	26 (22–31)	24 (21–28)	27 (23–33)	25 (22–31)	0.057	0.387
>20 cm^2^	103 (76.3)	33 (67.3)	26 (76.5)	77 (76.2)	0.367	0.978
Right Heart						
RV Dysfunction	2 (1.5)	1 (2.0)	0 (0.0)	2 (2.0)	1.000	1.000
PASP						
Median (mmHg)	39 (30–50)	32 (26–40)	44 (34–55)	38 (30–48)	0.001	0.115
≥50 mmHg	26 (19.3)	4 (8.2)	8 (23.5)	18 (17.8)	0.063	0.465

Data are presented as number (percentage), median (interquartile range), or mean ± standard deviation, where appropriate. EF = ejection fraction; EROA = effective regurgitant orifice area; ESD = end-systolic diameter; LA = left atrial; LV = left ventricular; MR = mitral regurgitation; PASP = pulmonary arterial systolic pressure; PISA = proximal isovelocity surface area; PVFP = pulmonary venous flow pattern; RV = right ventricular; RVol = regurgitant volume.

**Table 3:** Outcomes at 5 Years.

					*p*-Value	
	Total Cohort (N = 135)	Normal PVFP (N = 49)	Reversed PVFP (N = 34)	Non-Reversed PVFP (N = 101)	Normal vs. Reversed	Non-Reversed vs. Reversed
All-Cause Mortality, Mitral Intervention, or New-Onset Atrial Fibrillation	98 (72.6)	29 (59.2)	30 (88.2)	68 (67.3)	0.004	0.018
All-Cause Mortality	11 (8.1)	2 (4.1)	2 (5.9)	9 (8.9)	1.000	0.730
Mitral Intervention	87 (64.4)	25 (51.0)	29 (85.3)	58 (57.4)	0.001	0.003
New-Onset Atrial Fibrillation	22 (16.3)	4 (8.2)	4 (11.8)	18 (17.8)	0.711	0.408

Data are presented as number (percentage). PVFP = pulmonary venous flow pattern.

**Table 4:** Multivariable Cox Proportional Hazard Model for the Combined Outcomes of All-Cause Mortality, Mitral Intervention, or New-Onset Atrial Fibrillation at 5 Years.

	HR (95% CI)	*p*-Value
NYHA Class (per 1 class rise)	1.43 (1.09–1.87)	0.010
LV ESD ≥40 mm	2.11 (0.94–4.73)	0.069
LA Diameter (continuous)	1.47 (1.07–2.03)	0.018
RV Dysfunction	1.30 (0.17–9.92)	0.800
Severe MR	1.53 (0.85–2.75)	0.161
Posterior Prolapse Site	1.78 (0.98–3.23)	0.056
PVFP		
Abnormal (vs. Normal)	1.62 (0.93–2.84)	0.091
Reversed (vs. Normal)	2.53 (1.21–5.31)	0.011
Reversed (vs. Non-Reversed)	2.14 (1.12–4.10)	0.022

CI = confidence interval; EF = ejection fraction; ESD = end-systolic diameter; HR = hazard ratio; LA = left atrial; LV = left ventricular; MR = mitral regurgitation; NYHA = New York Heart Association; PVFP = pulmonary venous flow pattern; RV = right ventricular.

## Data Availability

The data underlying this article will be shared on reasonable request to the corresponding author.

## Supplementary Materials

The following supporting information can be downloaded at: https://www.mdpi.com/article/10.3390/jcdd10020049/s1, Figure S1: Five-Year Cumulative Incidence of the Combined Outcome of All-Cause Mortality, Mitral Intervention, or New-Onset Atrial Fibrillation According to Detailed Pulmonary Venous Flow Pattern at Baseline; Table S1: Univariable Cox Proportional Hazard Model for the Combined Outcome of All-Cause Mortality, Mitral Intervention, or New-Onset Atrial Fibrillation at 5 Years; Table S2: Cox Proportional Hazard Model for the Separate Outcomes at 5 Years.

## Abbreviations

LA = Left atrium

MR = Mitral regurgitation

dMR = Degenerative MR

PV = Pulmonary vein/venous

PVFP = Pulmonary venous flow pattern
