# Supplementary material for: The Prognostic Value of Pulmonary Venous Flow Reversal in Patients with Significant Degenerative Mitral Regurgitation
Source: J Cardiovasc Dev Dis. 2023 Jan 28;10(2):49. doi: 10.3390/jcdd10020049 (PMC9965059; doi:10.3390/jcdd10020049)
Supplement: Supplementary file 1 [file jcdd-10-00049-s001.zip › jcdd-2193545-supplementary.pdf]

Supplemental Figure S1. Five-Year Cumulative Incidence of the Combined Outcome of All-Cause Mortality, Mitral Intervention, or New-Onset Atrial Fibrillation According to Detailed Pulmonary Venous Flow Pattern at Baseline

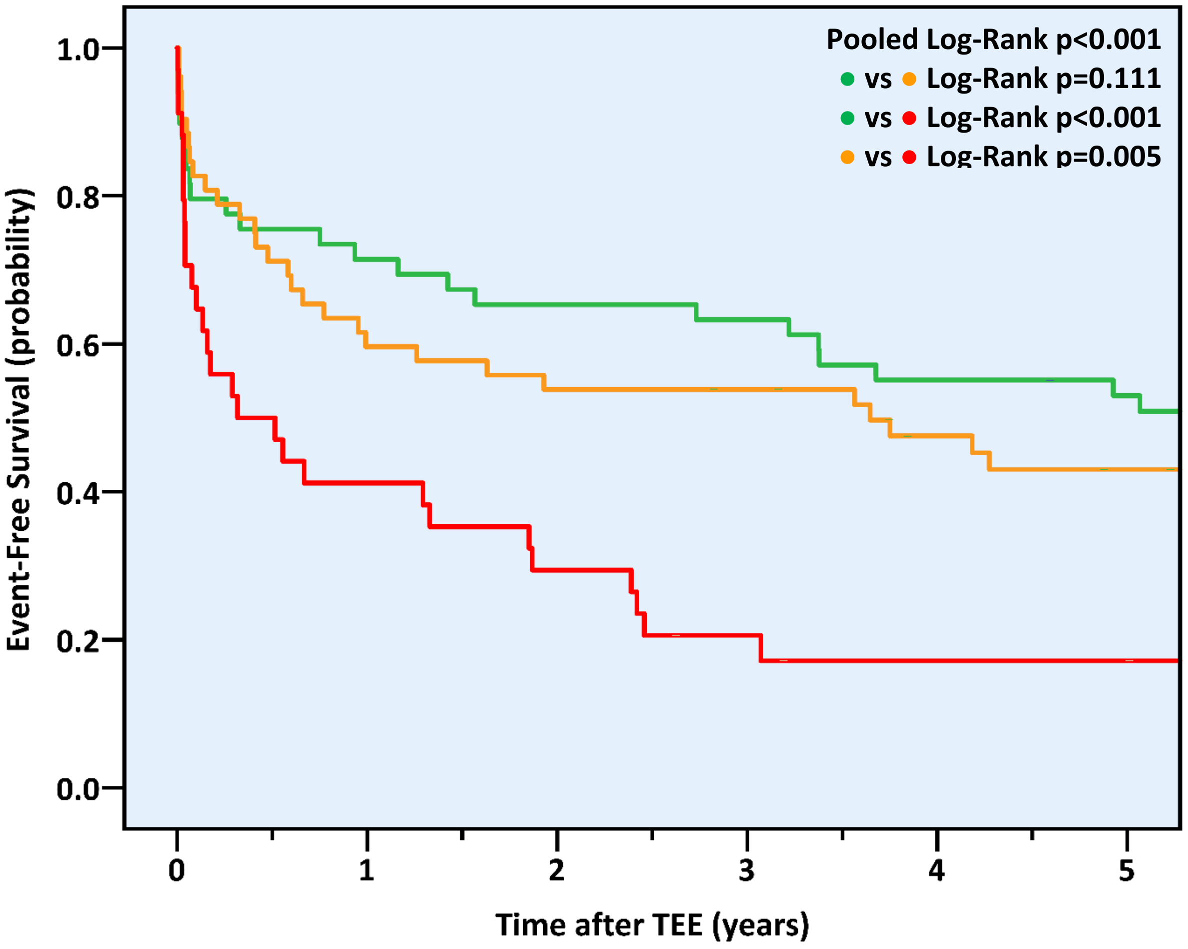

No. at Risk						
Normal PVFP	49	35	32	31	27	24
Blunted PVFP	52	31	28	26	21	18
Reversed PVFP	34	14	10	6	4	4

PVFP = pulmonary venous flow pattern; TEE = transesophageal echocardiogram

Supplemental Table S1. Univariable Cox Proportional Hazard Model for the Combined Outcome of All-Cause Mortality, Mitral Intervention, or New-Onset Atrial Fibrillation at 5 Years

	**HR (95% CI)**	**P-Value**
**Clinical Variables**		
Age (continuous)	0.99 (0.98-1.01)	0.475
Sex Male	1.04 (0.68-1.59)	0.859
BMI (continuous)	1.01 (0.96-1.06)	0.835
BSA (continuous)	1.18 (0.44-3.18)	0.746
Hypertension	0.73 (0.48-1.10)	0.133
Diabetes Mellitus	0.87 (0.43-1.72)	0.679
NYHA Class (per 1 class rise)	1.36 (1.05-1.78)	**0.021**
**Echocardiographic Parameters**		
Severe MR	1.99 (1.27-3.13)	**0.003**
MR PISA EROA		
Continuous	4.86 (1.28-18.41)	**0.020**
≥0.4cm^2	2.11 (1.06-4.21)	**0.033**
MR PISA RVol		
Continuous	1.01 (1.00-1.01)	0.064
≥60mL	1.71 (0.79-3.72)	0.175
Prolapse Site		
Anterior	0.49 (0.24-1.03)	0.059
Posterior	1.67 (1.06-2.63)	**0.028**
Both	0.78 (0,46-1.32)	0.359
LV ESD		
Continuous	0.99 (0.97-1.01)	0.318
≥40mm	0.56 (0.29-1.04)	0.068
LA Diameter		
Continuous	1.35 (1.02-1.79)	**0.015**
>55mm	1.48 (0.68-3.19)	0.325
LA Area		
Continuous	1.03 (1.01-1.050)	**0.017**
>20cm^2	1.07 (0.69-1.73)	0.770
RV Dysfunction	3.35 (0.82-13.73)	0.093

PASP		
Continuous	1.01 (0.99-1.03)	0.164
≥50mmHg	1.14 (0.69-1.86)	0.613
PVFP		**<0.001**
Reversed (vs Normal)	2.79 (1.66-4.70)	**<0.001**
Reversed (vs Non-Reversed)	2.28 (1.47-3.54)	**<0.001**

Figures in bold denote statistical significance.

BMI = body mass index; CI = confidence interval; EROA = effective regurgitant orifice area; ESD = end systolic diameter; HR = hazard ratio; LA = left atrial; LV = left ventricular; MR = mitral regurgitation; PASP = pulmonary arterial systolic pressure; NYHA = New York Heart Association; PISA = proximal isovelocity surface area; PVFP = pulmonary venous flow pattern; RV = right ventricular; RVol = regurgitant volume

Supplemental Table S2. Cox Proportional Hazard Model for the Separate Outcomes at 5 Years

	**All-Cause Mortality**				**Mitral Intervention**				**New-Onset Atrial Fibrillation**			
	**Univariable**		**Multivariable**		**Univariable**		**Multivariable**		**Univariable**		**Multivariable**	
	**HR (95% CI)**	**P-Value**	**HR (95% CI)**	**P-Value**	**HR (95% CI)**	**P-Value**	**HR (95% CI)**	**P-Value**	**HR (95% CI)**	**P-Value**	**HR (95% CI)**	**P-Value**
**Clinical Variables**												
Age (continuous)	1.08 (1.01-1.15)	0.027	1.07 (0.98-1.18)	0.145	0.99 (0.98-1.01)	0.405			1.04 (0.99-1.07)	0.070	1.03 (0.98-1.08)	0.261
Sex Male	1.94 (0.42-8.96)	0.398			1.26 (0.78-2.04)	0.336			0.72 (0.30-1.71)	0.456		
BMI (continuous)	1.16 (1.03-1.29)	0.011	1.26 (1.08-1.47)	**0.004**	1.01 (0.96-1.07)	0.675			1.02 (0.91-1.14)	0.726		
Hypertension	55.15 (0.33-92.51)	0.125			0.64 (0.42-0.99)	0.048	0.39 (0.22-0.67)	**0.001**	0.95 (0.41-2.21)	0.911		
Diabetes Mellitus	1.01 (0.13-7.94)	0.996			1.33 (0.67-2.66)	0.419			0.93 (0.22-3.96)	0.918		
NYHA Class (per 1 class rise)	2.19 (1.08-4.46)	0.031	2.36 (0.74-7.55)	0.148	1.59 (1.18-2.15)	0.002			1.39 (0.80-2.41)	0.245		
**Echocardiographic Parameters**												
Severe MR	0.91 (0.27-3.09)	0.873			1.97 (1.21-3.20)	0.006	1.51 (0.80-2.85)	0.205	5.63 (1.32-24.11)	0.020	6.21 (0.79-48.38)	0.081
MR PISA EROA												
Continuous	11.17 (0.59-21.85)	0.107			4.51 (1.10-18.36)	0.036			4.16 (0.68-25.60)	0.124		
≥0.4cm^2	1.55 (0.16-14.90)	0.705			2.12 (1.00-4.48)	0.050			7.09 (0.92-54.57)	0.060		
MR PISA RVol												
Continuous	1.00 (0.99-1.02)	0.448			1.00 (0.99-1.01)	0.299			1.00 (0.99-1.01)	0.401		
≥60mL	33.79 (0.03-95.20)	0.455			1.35 (0.61-2.97)	0.459			36.85 (0.22-62.34)	0.168		
Prolapse Site												
Anterior	0.75 (0.10-5.86)	0.784			0.55 (0.26-1.20)	0.134			0.36 (0.05-2.68)	0.319		
Posterior	1.22 (0.32-4.59)	0.773			1.59 (0.98-2.58)	0.062	2.21 (0.89-4.11)	0.102	1.64 (0.60-4.44)	0.334		

Both	0.92 (0.20-4.24)	0.910			0.77 (0.44-1.35)	0.363			0.85 (0.29-2.50)	0.763		
LV ESD												
Continuous	1.02 (0.95-1.08)	0.626			0.99 (0.97-1.02)	0.704			1.00 (0.96-1.05)	0.951		
≥40mm	2.09 (0.56-7.89)	0.276			0.60 (0.31-1.16)	0.126			0.79 (0.23-2.67)	0.706		
LA Diameter												
Continuous	1.26 (0.57-2.79)	0.572			1.16 (0.86-1.55)	0.331			1.34 (0.74-2.42)	0.328		
>55mm	4.15 (0.90-19.28)	0.069			0.91 (0.37-2.25)	0.838			0.84 (0.11-6.22)	0.861		
LA Area												
Continuous	1.04 (0.98-1.10)	0.251			1.02 (0.99-1.05)	0.117			1.06 (1.02-1.11)	0.006	1.06 (1.03-1.12)	**0.038**
>20cm^2	1.40 (0.30-6.48)	0.667			1.21 (0.67-1.86)	0.661			3.40 (0.80-14.56)	0.099		
RV Dysfunction	0.05 (0.00-92.33)	0.774			2.47 (0.61-10.09)	0.207			33.34 (6.88-61.70)	<0.001	10.54 (1.17-94.99)	**0.036**
PASP												
Continuous	1.02 (0.98-1.07)	0.342			1.00 (0.99-1.02)	0.642			1.04 (1.01-1.06)	0.012	0.99 (0.96-1.04)	0.964
≥50mmHg	0.43 (0.06-3.33)	0.417			1.01 (0.59-1.73)	0.981			2.22 (0.90-5.44)	0.082		
PVFP												0.051
Reversed (vs Normal)	1.60 (0.23-11.36)	0.639			2.78 (1.61-4.81)	<0.001	1.98 (1.03-4.35)	**0.009**	1.55 (0.39-6.21)	0.534		
Reversed (vs Non-Reversed)	0.70 (0.15-3.23)	0.697			2.24 (1.42-3.54)	0.001	1.58 (1.35-2.97)	**0.016**	0.64 (0.22-1.89)	0.419		
**Competing Risks**												
Mitral Intervention Before AF	0.44 (0.14-1.45)	0.443			NA	NA			0.63 (0.27-1.45)	0.275		
New-Onset AF Before Mitral Intervention	1.07 (0.23-4.94)	0.933			0.54 (0.22-1.32)	0.175			NA	NA		

Figures in bold denote statistical significance.

AF = atrial fibrillation; BMI = body mass index; CI = confidence interval; EROA = effective regurgitant orifice area; ESD = end systolic diameter; HR = hazard ratio; LA = left atrial; LV = left ventricular; MR = mitral regurgitation; MRA = mineralocorticoid receptor antagonist; NYHA = New York Heart Association; PASP = pulmonary arterial systolic pressure; PISA = proximal isovelocity surface area; PVFP = pulmonary venous flow pattern; RV = right ventricular; RVol = regurgitant volume
